# Altered Carbohydrates Allocation by Associated Bacteria-fungi Interactions in a Bark Beetle-microbe Symbiosis

**DOI:** 10.1038/srep20135

**Published:** 2016-02-03

**Authors:** Fangyuan Zhou, Qiaozhe Lou, Bo Wang, Letian Xu, Chihang Cheng, Min Lu, Jianghua Sun

**Affiliations:** 1 State Key Laboratory of Integrated Management of Pest Insects and Rodents, Institute of Zoology, Chinese Academy of Sciences, Beijing, 100101, China; 2 University of Chinese Academy of Sciences, Beijing, 100049, China; 3 Technical Center, Hebei Entry-Exit Inspection and Quarantine Bureau, Shijiazhuang, 050051, China; 4 Key Laboratory of Tropical Forest Ecology, Xishuangbanna Tropical Botanical Garden, Chinese Academy of Sciences, Menglun, 666303, China

## Abstract

Insect-microbe interaction is a key area of research in multiplayer symbiosis, yet little is known about the role of microbe-microbe interactions in insect-microbe symbioses. The red turpentine beetle (RTB) has destroyed millions of healthy pines in China and forms context-dependent relationships with associated fungi. The adult-associated fungus *Leptographium procerum* have played key roles in RTB colonization. However, common fungal associates (*L. procerum* and *Ophiostoma minus*) with RTB larvae compete for carbohydrates. Here, we report that dominant bacteria associated with RTB larvae buffer the competition by inhibiting the growth and D-glucose consumption of *O. minus*. However, they didn’t inhibit the growth of *L. procerum* and forced this fungus to consume D-pinitol before consuming D-glucose, even though D-glucose was available and a better carbon source not only for *L. procerum* but also for RTB larvae and associated bacteria. This suggests the most frequently isolated bacteria associated with RTB larvae could affect fungal growth and the sequence of carbohydrate consumption. Thus, this regulates carbohydrate allocation in the RTB larva-microbe community, which may in turn benefit RTB larvae development. We also discuss the mechanism of carbohydrate allocation in the RTB larva-microbe community, and its potential contribution to the maintenance of a symbiotic community.

In insect-microbe symbioses, microbial associates consist of multiple-taxa, including bacteria, filamentous fungi, and yeasts, which form a complex and interacting network^1^. Much attention has been focused on the effects of specific species of microbial associates on the insect host, and both beneficial and detrimental aspects have been reported. For beneficial microbes, in addition to providing nutritional supply^2^,^3^, microbes can also protect insects against plant allelochemicals^4^,^5^, predators^6^ as well as parasitoids^7^. Detrimental microbes are reported as a source of infectious disease^8^,^9^ and they may also compete for nutrition with the host insect^10^. Except for the bilateral frameworks between the insect host and one class of microbial associates mentioned above, few studies have investigated how microbial associates may interact in their shared niches. It is becoming evident that microbe-microbe interactions in insect-microbe symbioses are strong^11^,^12^ and these interactions are thought to shape the microbial community structure and ultimately affect the overall results of insect-microbe interactions^1^.

Bark beetle-fungus symbioses have been the classic model system for studying insect-fungus interactions^13^, in which multiple effects of associated fungi on bark beetles such as nutrition contribution^14^,^15^, pheromone production^16^, and overcoming tree defense^17^,^18^ as well as saccharide competition^10^ are revealed. Besides, a myriad of bacteria have also been isolated from bark beetles, and they are reported to play roles in transforming beetle pheromones^16^, improving insect nutrition^19^,^20^, and defending against detrimental factors^21^,^22^ for beetles. Pair-wise interactions between bark beetle-associated bacteria and fungi, especially the effects of bacteria on fungi (both positive and negative), have been reported to limit fungal growth and reproduction^23^,^24^,^25^, but the roles of bacteria-fungi interactions in bark beetle-microbe symbiosis have not yet been revealed.

The red turpentine beetle (RTB), *Dendroctonus valens* LeConte (Coleoptera: Curculionidae: Scolytinae), has colonized and killed at least 10 million healthy pine *Pinus tabuliformis* Carrière in China^26^. Various blue-stain fungi including *Leptographium procerum*, *Leptographium sinoprocerum* and *Ophiostoma minus* have been isolated from healthy RTB larvae, while severe antagonistic relationships between these 3 fungal species and RTB larvae resulting from saccharide competition have been revealed in laboratory experiments^10^. The negative effects of these frequently associated fungi on bark beetle larva growth pose as risks to the survival of the beetle larvae. Thus growth of these fungi have to be controlled in order to enhance beetle survival^13^,^27^. In addition to the fungi, many bacteria are also brought inside galleries and become associated with RTB larvae and sealed inside with frass. Thus, it provides us a suitable model to look into associated bacteria-fungi interactions and their potential effects on host insects and themselves in the context of bark beetle-microbe symbiosis.

Associated fungi can compete with RTB larvae for carbohydrates^10^, while associated bacteria can affect growth of some fungi associated with bark beetles^25^. Thus, we hypothesized that by regulating the growth or carbohydrate consumption of associated fungi, associated bacteria may attenuate the competition for saccharides between RTB larvae and associated fungi and indirectly mediate the carbohydrate allocation in this symbiosis. To test the hypothesis, bacteria were isolated throughout the RTB larval period in China using a culture-dependent method, and their effect on the RTB-fungi interactions was investigated. Secondly, a series of bioassays were conducted to test the influence of the most frequently isolated bacteria on the growth and carbohydrate consumption of two common fungi associated with RTB larvae, *L. procerum* and *O. minus*. Lastly, effects of main carbohydrate components in phloem medium on the growth of the fungus *L. procerum*, RTB larvae and frequently isolated bacteria were analyzed.

## Results

### Bacterial species isolated from RTB larvae

In total, 1,088 bacterial strains were isolated from the gut, surface and frass of 1^st^ instar to 5^th^ instar RTB larvae (Supplementary, Table S1 online), among which 77 bacterial strains were selected and sequenced (Supplementary, Table S2 online). Twenty-two bacterial species belonging to 13 genera in 8 families in 3 phyla were identified by combination of BLAST search and phylogenetic analyses (Supplementary, Table S2 and Fig. S1 online). Phylogenetic analyses (in addition to blast search, morphology and isolation source) determined that bacterial strain B301, which was closely related to both *Serratia liquefaciens* and *Rahnella aquatilis*, was actually *R. aquatilis* (Supplementary, Fig. S1 online). Morphologically similar bacterial strain B321, B322, and B911 were identified as *Pseudomonas* sp.7 as they clustered together on the same phylogenetic tree with a high degree of similarity (>99.4%) to *P. brenneri* CFML 97–391 (AF268968) but didn’t group with any type strain well.

**Figure 1:**
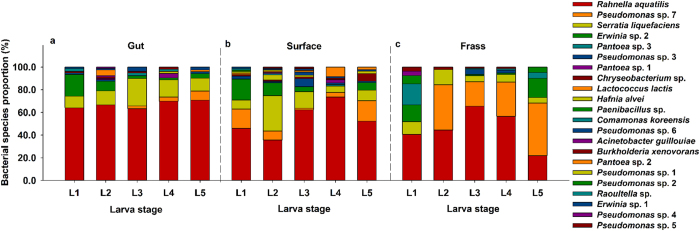
Composition of bacterial communities from gut (**a**), surface (**b**) and frass (**c**) of RTB larvae at different developmental stages. Bar value was represented by isolation frequency (%) of each bacterial species in corresponding isolation source. “L1” to “L5” referred to RTB larvae ranged from 1^st^ instar to 5^th^ instar. Original data were provided in Supplementary, Table S1 online.

Isolation frequencies of *R. aquatilis* (630/1,088), *S. liquefaciens* (135/1,088) and *Pseudomonas* sp. 7 (134/1,088) from each RTB larval stage from all isolation sources were higher than those of other bacteria species (Fig. 1, Supplementary, Table S1 online). Amongst these 3 bacterial species, isolation frequency of *R. aquatilis* (630/1,088) was the highest (Fig. 1, Supplementary, Table S1 online). Eight bacterial strains (*R. aquatilis* B301 B302 B904, *S. liquefaciens* B310 B317, *Pseudomonas* sp. 7 B321 B322 B911) from the 3 most frequently isolated bacterial species were used in subsequent experiments.

### Bacteria alleviate the antagonistic effects of fungi *L. procerum* (CMW25626) and *O. minus* (CMW25254) on RTB larva growth

**Figure 2:**
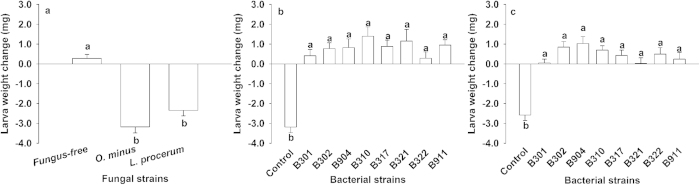
Bacteria alleviate the antagonistic effects of fungi *O. minus* (CMW25254) and *L. procerum* (CMW25626) on the weight change of RTB. Average weight change of RTB larvae during a 6-day period after feeding on: (**a**) the control (sterile medium) and the medium with one of two fungal species (*O. minus* and *L. procerum*, n = 36 for each treatment), (**b**) the control (*O. minus*-colonized medium) and *O. minus*-colonized medium in the presence of bacterial strains (*R. aquatilis* B301 B302 B904, *S. liquefaciens* B310 B317, *Pseudomonas* sp. 7 B321 B322 B911, n = 40 for each treatment), (**c**) the control (*L. procerum*-colonized medium) and *L. procerum*-colonized medium in the presence of the eight bacterial strains (n = 40 for each treatment). Data show mean + s.e.m. Different letters within each group of bars refer to significant difference of multiple comparisons by Tukey method for Fig. 2a, Dunnett’s T3 test method for Fig. 2b,c.

RTB larva weight decreased significantly on *L. procerum*-colonized phloem media and *O. minus*-colonized phloem media compared to fungus-free phloem media (Fig. 2a, one-way ANOVA, *F*_2,117_ = 45.070, *p* < 0.001). Weight change of RTB larvae in phloem media colonized by *O. minus* in the presence of the eight bacterial strains (*R. aquatilis* B301 B302 B904, *S. liquefaciens* B310 B317, and *Pseudomonas* sp. 7 B321 B322 B911) were significantly higher than that in *O. minus*-grown phloem media alone (Fig. 2b, Welch’s ANOVA test, *F*_8,145.881_ = 19.951, *p* < 0.001). Similarly, weight change of RTB larvae in phloem media colonized by *L. procerum* in the presence of the eight bacterial strains were significantly higher than those feeding on *L. procerum*-grown phloem media alone (Fig. 2c, Welch’s ANOVA test, *F*_8,145.876_ = 14.308, *p* < 0.001).

### Different responses of growth and carbohydrate consumption in two fungi to dominant bacteria species

**Figure 3:**
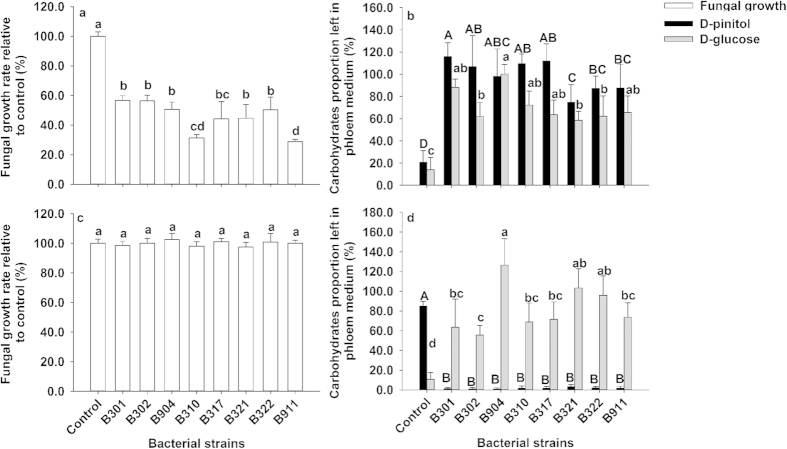
Effects of the most frequently isolated bacterial strains on the growth and carbohydrate consumption of *O. minus* (CMW25254) and *L. procerum* (CMW25626) on phloem media. (**a**) Linear growth of *O. minus* (CMW25254) in the presence of the most frequently isolated bacterial strains (*R. aquatilis* B301 B302 B904, *S. liquefaciens* B310 B317, *Pseudomonas* sp. 7 B321 B322 B911, n = 5 for each treatment). (**b**) Carbohydrate consumption of *O. minus* (CMW25254) on phloem media in the presence of the most frequently isolated bacterial strains (n = 5 for each treatment). (**c**) Linear growth of *L. procerum* (CMW25626) in the presence of the most frequently isolated bacterial strains. (**d**) Carbohydrate consumption of *L. procerum* (CMW25626) on phloem media in the presence of the most frequently isolated bacterial strains (n = 5 for each treatment). Fungal growth on fungus-grown phloem media was represented as change of mycelia linear growth rate relative to control (+s.e.m.). Carbohydrate consumption in fungus-grown phloem media was represented as carbohydrate content left relative to fungus-free phloem media (+s.e.m.). Different letters above each bar referred to significant difference of multiple comparisons by Tukey method within each set of bars in Fig. 3a–d (*capital letters* for D-pinitol in Fig. 3b,d, and *lowercase letters* for fungal growth in Fig. 3a,c and D-glucose in Fig. 3b,d).

The growth of *O. minus* (CMW25254) was significantly inhibited by the eight bacteria strains associated with RTB (*R. aquatilis* B301 B302 B904, *S. liquefaciens* B310 B317, and *Pseudomonas* sp. 7 B321 B322 B911, Fig. 3a, one-way ANOVA, *F*_8,36_ = 52.780, *p* < 0.001). Its mycelial linear growth was significantly decreased to about 60% relative to control by the eight bacterial strains. However, linear growth of *L. procerum* (CMW25626) was not significantly affected by the bacterial strains as shown in Fig. 3c (one-way ANOVA, *F*_8,36_ = 1.084, *p* = 0.396).

For *O. minus* (CMW25254), consumption of D-pinitol (one-way ANOVA, *F*_8,36_ = 28.608, *p* < 0.001) and D-glucose (one-way ANOVA, *F*_8,36_ = 8.974, *p* < 0.001) on phloem media were significantly reduced by eight bacterial associates of RTB. Particularly, at least 60% of D-pinitol and D-glucose respectively remained unconsumed in the presence of the eight bacteria after 15d of *O. minus* (CMW25254) growth in phloem media, while without the eight bacteria, 20.8% of D-pinitol and13.9% of D-glucose remained unconsumed relative to controls (Fig. 3b). For *L. procerum* (CMW25626), consumption of D-pinitol (one-way ANOVA, *F*_8,36_ = 737.150, *p* < 0.001) and D-glucose (one-way ANOVA, *F*_8,36_ = 14.499, *p* < 0.001) on phloem media was significantly affected by the eight associated bacterial strains (Fig. 3d). Specifically, almost all D-pinitol in phloem was consumed up by *L. procerum* (CMW25626) in all bacterial treatments, while about 85.0% of D-pinitol remained unconsumed in fungus-grown phloem media in the absence of the eight bacterial strains. On the contrary, at least 55.5% of D-glucose remained unconsumed after fungi growth in each bacterial treatment, while there were only about 10.7% of D-glucose unconsumed in the fungus-grown phloem media in the absence of the eight associated bacterial strains.

### Similar response of growth and carbohydrate consumption sequence in *L. procerum* (CMW25626) to different amounts of *R. aquatilis* B301 cultures

There was no significant difference in mycelial linear growth rate of *L. procerum* (CMW25626) among different amounts of *R. aquatilis* B301 culture treatments (Supplementary, Fig. S2a, one-way ANOVA, *F*_4,30_ = 1.052, *p* = 0.397, online). As for carbohydrates, D-pinitol (Supplementary, Fig. S2b, one-way ANOVA, *F*_4,30_ = 968.759, *p* < 0.001, online) and D-glucose (Supplementary, Fig. S2b, one-way ANOVA, *F*_4,30_ = 52.112, *p* < 0.001, online) consumption were significantly affected by the amount of bacterial culture added. D-pinitol content in fungus-grown phloem in 1-drop, 3-drop, 5-drop and 100 μL-spread bacterial treatment was decreased to 8.7%, 4.4%, 4.0% and 3.3% relative to fungus-free phloem medium, while 116.2% remained unconsumed in the control group. Instead, D-glucose remained unconsumed in amounts 124.0%, 113.3%, 87.8% and 86.1% relative to fungus-free phloem media in 1-drop, 3-drop, 5-drop and 100 μL-spread treatments, respectively, while only 40.2% of D-glucose remained unconsumed in the control group.

### Altered carbohydrate consumption sequence in *L. procerum* (CMW25626) in the presence of bacteria *R. aquatilis* B301

In the bacteria-free control group, content of D-pinitol (Supplementary, Fig. S3a, one-way ANOVA, *F*_6,35_ = 19.961, *p* < 0.001, online) and D-glucose (Supplementary, Fig. S3a, one-way ANOVA, *F*_6,35_ = 217.182, *p* < 0.001, online) decreased significantly over time in phloem media following *L. procerum* (CMW25626) growth. Particularly, D-glucose in phloem media inoculated with the fungal strain began to decrease significantly at 5d, while D-pinitol didn’t show any significant decrease until 20d. In bacterial treatment groups, content of D-pinitol (Supplementary, Fig. S3b, one-way ANOVA, *F*_6,35_ = 500.035, *p* < 0.001, online) and D-glucose (Supplementary, Fig. S3b, one-way ANOVA, *F*_6,35_ = 115.910, *p* < 0.001, online) also decreased significantly over time in phloem media following *L. procerum* (CMW25626) growth. Specifically, D-glucose was not consumed significantly until 15d, while D-pinitol began to be consumed at 5d.

### D-glucose is the better carbon source for *L. procerum* (CMW25626), eight commonly associated bacterial strains and RTB larvae compared to D-pinitol

**Figure 4:**
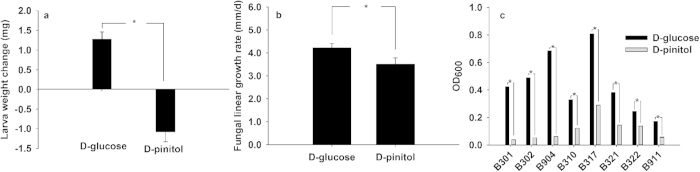
Effects of D-pinitol and D-glucose on the growth of *L. procerum*, RTB larva, and the most frequently isolated bacterial strains. (**a**) The linear growth rate of *L. procerum* on the media which contained only D-pinitol or D-glucose as carbon source respectively (growth rate, mm/d, +s.e.m., n = 9 for each treatment). (**b**) Weight change of larvae feeding on phloem media containing D-pinitol or D-glucose respectively (weight change, mg, +s.e.m., n = 56 for each treatment). (**c**) The growth of the most frequently isolated bacterial strains in liquid cultures which contained D-pinitol or D-glucose respectively (OD_600_, +s.e.m., n = 3 for each treatment). “*” between the two connected bars referred to significant difference.

Mycelial growth rate of *L. procerum* (CMW25626) on agar plates containing D-glucose was significantly higher than that on agar plates containing D-pinitol (Fig. 4a, *df* = 16, *t* = 6.448, *p* < 0.001). RTB larvae weight change in phloem media with D-glucose was significantly higher than that in phloem media with D-pinitol at 6d (Fig. 4b, *df* = 101.080, *t* = −7.410, *p* < 0.001). Absorbance at 600 nm of each of the eight bacterial strains (*R. aquatilis* B301 B302 B904, *S. liquefaciens* B310 B317, and *Pseudomonas* sp. 7 B321 B322 B911) in liquid culture containing D-glucose was significantly higher than that containing D-pinitol (Fig. 4c; B301, *df* = 4, *t* = 304.797, *p* < 0.001; B302, *df* = 4, *t* = 824.087, *p* < 0.001; B904, *df* = 4, *t* = 166.800, *p* < 0.001; B310, *df* = 4, *t* = 3124.000, *p* < 0.001; B317, *df* = 4, *t* = 354.133, *p* < 0.001; B321, *df* = 4, *t* = 376.654, *p* < 0.001; B322, *df* = 4, *t* = 913.657, *p* < 0.001; B332, *df* = 4, *t* = 56.824, *p* < 0.001).

## Discussion

**Figure 5:**
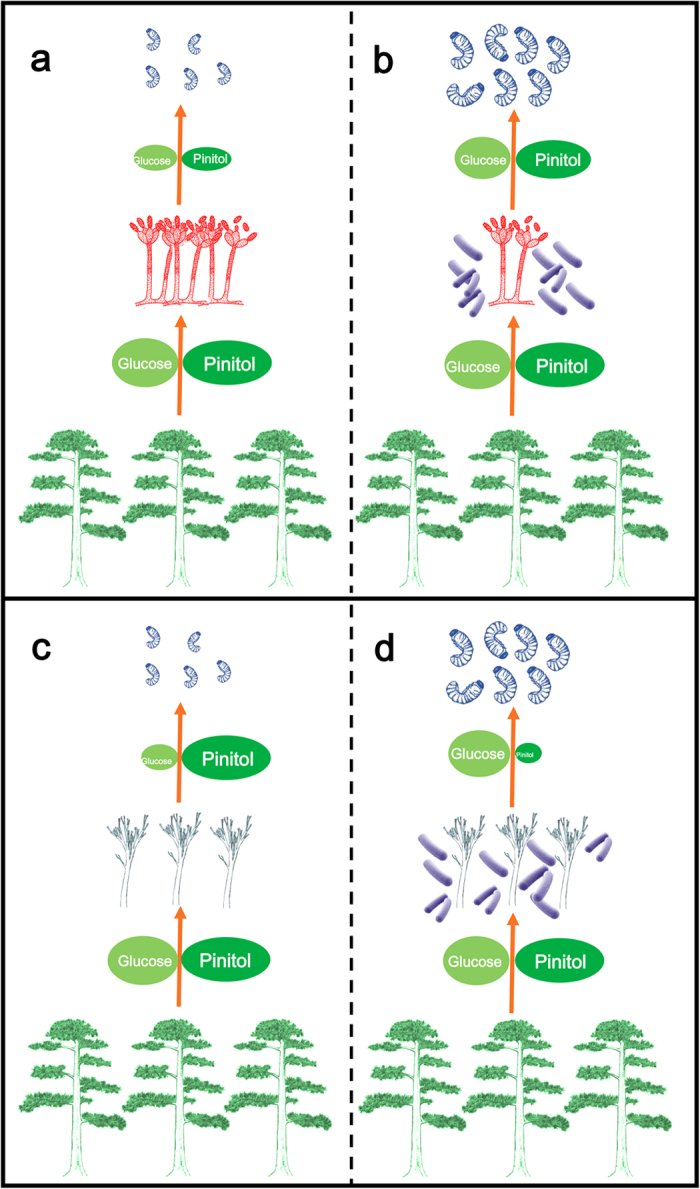
Associated bacteria mediate carbohydrate allocation in RTB larva-microbe community. (**a**) The fungus *O. minus* consumes most of the D-pinitol and D-glucose in phloem in the absence of associated bacteria. (**b**) In the presence of bacteria, the growth of the fungus is inhibited, and D-glucose and D-pinitol remain unconsumed and is left for RTB larvae. (**c**) The fungus *L. procerum* consumes D-glucose before consuming D-pinitol in the phloem in the absence of associated bacteria. (**d**) The fungus *L. procerum* consumes D-pinitol before consuming D-glucose in the presence of bacteria, and D-glucose, a better carbohydrate compared to D-pinitol, remains unconsumed and is left for RTB larvae.

Our work provides laboratory evidence that bacteria-fungi interactions mediate carbohydrate allocation in two ways in RTB larva-microbe symbiosis (Fig. 5), which could attenuate the competition for saccharides between the RTB larva and its associated fungi (Fig. 2). On one hand, the most frequently isolated bacteria *R. aquatilis*, *S. liquefaciens* and *Pseudomonas* sp. 7, which are commonly isolated from *D. valens* in North America and Mexico^19^,^20^,^28^, inhibit *O. minus* growth (Fig. 3a), resulting in more carbohydrates remaining for RTB larvae after fungal growth on phloem media (Fig. 3b). On the other hand, these bacteria don’t affect the growth of *L. procerum* (Fig. 3c), but instead alter the carbohydrate consumption sequence of *L. procerum* on phloem media (Fig. 3d), and the effects of these associated bacteria are dose-independent (Supplementary, Fig. S2 online). In the absence of these commonly associated bacteria, *L. procerum* consumes D-glucose in priority of D-pinitol (Supplementary, Fig. S3a online), while in the presence of these bacteria, it consumes D-pinitol in priority of D-glucose (Supplementary, Fig. S3b online). D-glucose is a better carbon source in terms of RTB larval weight gain, fungal growth as well as the bacterial growth compared to D-pinitol (Fig. 4). Briefly, in the presence of associated bacteria, more D-glucose remained unconsumed and available for RTB larvae than in the absence of associated bacteria.

Associated bacteria play key roles in initiating carbohydrate allocation in RTB larvae-microbe symbiosis. The associated bacteria may influence the fungal growth and carbohydrate consumption in the following three ways. First, as previous reported, volatiles derived from bark beetle-associated bacteria could affect fungal growth^25^. These volatiles may also affect the sequence of carbohydrate consumption by the two fungi in our study. Second, the associated bacteria could convert (+)-α-pinene to the terpene oxides, just like cis-verbenol and verbenone^22^ which have been reported to inhibit linear growth of *L. procerum* and *O. minus*^29^, or even alter the carbohydrate consumption in the fungus. Third, (+)-α-pinene may affect the metabolic pathways^30^ or volatile profiles of the associated bacteria, and the (+)-α-pinene-induced bacterial metabolic pathways may influence the growth and carbon consumption of the fungi. Volatiles derived from different bacterial strain vary both qualitatively in composition and quantitatively in the ratios in which they occur^31^. (+)-α-pinene could also affect the metabolic pathways in bacteria that synthetize volatiles. Thus, the volatile composition in this experiment becomes complicated, and further research needs to be conducted to test the individual components of the volatiles using metabolomics and to test the effects of each component on fungal growth and carbohydrate consumption sequence.

The fungus *L. procerum* is another participant in mediating carbohydrate allocation in RTB larva-microbe symbiosis, and its consumption sequence for D-pinitol and D-glucose is altered. Generally, utilization of secondary favored substrates are prevented in the presence of the preferred substrates in microorganisms, which is called carbon catabolite repression (CCR)^32^. The most common example for CCR is the glucose effect in which consumption of other carbon sources is repressed until glucose is consumed by bacteria in culture broth^33^. Our results show *L. procerum* consumed D-glucose before D-pinitol on phloem media in the absence of bacteria, and conforms to CCR, while the phenomenon that this fungus consumed D-pinitol before D-glucose in the presence of bacteria is little known. Bacteria-derived volatiles are comprised of various organic acids^31^, and some of them could be consumed in priority of glucose by several bacterial genera, such as *Azospirillum*, *Arthrobacter*, *Pseudomonas*, and *Rhizobium* bacteria^34^, and thus might inhibit the consumption of D-glucose in *L. procerum*, while the consumption of D-pinitol is not affected. However, further qualitative experiments showed that the associated bacteria didn’t significantly suppress the growth of *L. procerum* on medium containing D-glucose as the sole carbon source (Data not shown), indicating that D-glucose consumption was not inhibited. Another possible explanation for this phenomenon is that the associated bacteria may affect the CCR pathway in *L. procerum*. CCR pathway and laccase synthesis have been proven to be correlated in filamentous fungi^35^, and laccase activity of the fungus, *Phanaerochaete magnoliae*, is completely ceased by volatiles from all selected bacterial strains^36^, which implies that volatiles may affect the CCR pathway as well. Further experiments on transcription and translation of key genes in the CCR pathway need to be conducted to clarify the mechanism of the altered carbohydrate consumption sequence in the fungus *L. procerum*.

Carbohydrate allocation mediated by bacteria-fungi interactions may have profound effects on the relationship between the RTB larva and its associated fungi, and these effects may have extending influence on the symbiont abundance and the symbiosis community structure in RTB larva-microbe symbiosis. A smaller number of carbohydrates were allocated to the detrimental fungal symbiont *O. minus* and the growth of this fungus was inhibited by bacterial associates. Thus, the abundance of the fungus would likely decrease during larval development, resulting in larval protection. Surprisingly, for the context-dependent beneficial fungus *L. procerum*, D-pinitol, the less preferred carbohydrate, was allocated to the fungus instead of the preferred carbohydrate, D-glucose, but its growth was not affected (Fig. 3c), which may indicate an adaption of the fungus to the gallery environment. Maybe it is this adaptation that makes this fungus to be the dominant fungus in the gallery in different developmental stages of RTB, which was reported in previous samplings^10^,^37^. The carbohydrate D-pinitol consumed by *L. procerum*, reported to be detrimental to *Heliothis zea*^38^, *Aedes aegypti*^39^, and *Culex quinquefasciatus*^39^, also inhibited the growth of RTB larvae in this study. Consumption of D-pinitol by *L. procerum* in the presence of associated bacteria indicates a mutualistic relationship between the fungus and RTB larvae. In the context of bacteria-fungi interactions, D-glucose, the most preferred carbon source, was allocated to RTB larvae, which could allow the persistence of the symbiosis. Analogous to mycorrhizal symbioses in which photosynthate of the plant is preferentially allocated to the beneficial fungal symbiont^40^, the redistribution of carbon resources in this microbial-insect symbiosis might also stabilize the mutualism. As RTB larvae growth was not affected by its associated bacteria in non-contact bioassays (Supplementary, Fig. S4 online), re-distribution of carbon sources in this community might support their survival. Surviving larvae in turn could provide habitats and act as vectors for microbes. The associated bacteria play key roles in regulating the symbiotic relationship and they may also benefit from altered carbohydrate allocation, for D-glucose is also their preferred carbon source compared to D-pinitol (Fig. 4c). Associated bacteria consume D-glucose, which seems to result in competition between bacteria and RTB larvae for D-glucose. In fact, D-glucose consumed by bacteria may be utilized for construction of the symbiosis, i.e., the cost of mutualism^41^. This mediated carbohydrate allocation may structure the symbiotic community, as it has been previously reported in plant-arbuscular mycorrhizal fungi symbiosis^40^,^41^. Our work has its limitations since using artificial growth media and nutrient supplies may influence bacterial metabolism or volatile profile^42^,^43^, but the *in vitro* bioassays with standard laboratory conditions might be the best option because of difficulties in performing experiments in uncontrolled bacteria-fungi interactions in the field. In addition, the extent to which bacteria-fungi interactions contribute to mediation of carbohydrate allocation needs to be quantified by further experiments that focus on excluding bacteria in the bark beetle larva-microbe community.

Symbiotic microbes have been reported to play both positive and negative roles in shaping multiple aspects of insect biology and phenotype^1^,^10^,^44^. However, studies linking microbe-microbe interactions to insect-microbe interactions are surprisingly few, given that a large number of phytophagous species harbor a myriad of microbes of different taxonomic clades^1^. The microbe-microbe interaction may affect pair-wise interactions between the insect and microbial associates. Our work is another case study that reveals the massive importance of microbe-microbe interactions in understanding symbiotic relationships and provides evidence that inter-specific interactions can be regulated accurately by mediation of carbohydrate allocation. Further work needs to test microbe-microbe interactions among the broad taxonomic range of insect-microbe symbioses.

## Methods

### Experiment I Bacteria isolation and identification

RTB larvae and frass samples were collected from Tunlanchuan Forestry Farm (N 37°48′ E 111°44′, average elevation 1400 m, Shanxi, China). Detailed information on sample collection, bacteria isolation and identification procedures are provided in Supplementary online.

### Experiment II Effects of fungus-colonized phloem media in the presence or in the absence of bacteria on RTB larval growth

To test whether the bacterial strains could alleviate or compromise the antagonistic effects of fungi *O. minus* and *L. procerum* on RTB larval growth, we first investigated the effects of fungus-colonized phloem media on RTB larval growth according to the method described previously^10^. Briefly, weight changes of RTB larva feeding on medium with a specific fungal strain and without fungal strain (control) were compared. Each treatment had 36 replicates. Second, weight changes of RTB larva feeding on phloem medium colonized by specific fungus in the absence (control) or presence of each of eight bacterial strains during fungal growth were compared. The bacteria-free group was regarded as controls. Each treatment had 40 replicates. Detailed methods are provided in Supplementary online.

### Experiment III Effects of the most frequently isolated bacteria on growth and carbohydrate consumption of two fungi on phloem media

Two fungal strains, *L. procerum* (CMW25626) and *O. minus* (CMW25254), were selected as they are commonly associated with RTB larvae and both fungal strains compete for D-fructose and D-glucose with RTB larvae^10^. Except D-fructose and D-glucose, D-pinitol is also dominant carbohydrate in *P. tabuliformis* phloem and phloem media according to Wang^10^ and in our studies (Supplementary, Fig. S5, Table S3 online). A preliminary experiment showed that associated bacterial strains (B301, B310 and B911) inhibited *O. minus* growth (Supplementary, Fig. S6a, online) and its consumption of D-glucose, D-pinitol and D-fructose (Supplementary, Fig. S6b, online). Dissimilarly, these strains didn’t inhibit growth of *L. procerum* (Supplementary, Fig. S6c, online), but they affected its consumption of D-glucose and D-pinitol (Supplementary, Fig. S6d, online). As the consumption of D-fructose in *L. procerum* (CMW 25626) on phloem media was not affected by different bacterial treatments (Supplementary, Fig. S6d, online), its content wasn’t detected in the following experiments.

The eight most frequently isolated bacterial strains (*R. aquatilis* B301 B302 B904, *S. liquefaciens* B310 B317, *Pseudomonas* sp. 7 B321 B322 B911) were selected for further tests with the same arena (Supplementary online). A 90 mm Petri dish, separated into two parts with a plastic baffle plate, was used. Briefly, LBA was poured into one side of the petri dish, and phloem medium was poured into the opposite side. Bacterial and fungal cultures were inoculated onto corresponding media. Pine monoterpenes, particularly (+)-α-pinene, have been reported to amplify, reduce, or reverse the effects of bacteria on fungi in bark beetle microbial community^25^, thus the dominant pine volatile (+)-α-pinene^45^ was added into the arena to simulate the pine chemical environment. The petri dishes were sealed with teflon. Bacteria-free LBA plate with (+)-α-pinene was used as control, and each treatment was replicated 5 times. Fungal linear growth was measured every 2d, and carbohydrate composition unconsumed in phloem media was measured 15d later when the RTB are 1^st^ instar larvae and the fungal hyphae covered 100% of the medium surface, and data were presented as percent proportion relative to control.

### Experiment IV Effects of different amounts of *R. aquatilis* B301 cultures on *L. procerum* growth and carbohydrate consumption sequence on phloem media

To test whether the effects from bacteria on *L. procerum* were caused by an unreasonable amount of bacterial treatment, various amounts of bacterial cultures were used in further experiments using methods previously described by Blom *et al*.^43^. Only one bacterial strain, *R. aquatilis* B301, was used in this experiment as the isolation frequency of this species was the highest (Fig. 1). Other steps were identical to the arena described in Supplementary online. OD_600_ of actively growing *R. aquatilis* B301 cultures in LB was tested (≈0.5) to detect the initial cell quantity in the cultures, and 0 (control), 1, 3, 5 drops (20 μL/drop) of bacterial cultures were dropped on LBA plate with a 20 μL micropipettor to simulate different amounts of bacteria. Individual drops of bacterial cultures didn’t contact with each other. In addition, 100 μL of bacterial cultures were coated on LBA plate for comparison to the 5-drop treatment. Fungal linear growth rate, D-glucose and D-pinitol content in fungus-grown phloem media at 15d were tested as described in Supplementary online. Each treatment was replicated 7 times. Fungal linear growth and carbohydrate composition were measured, and data were presented as the percent proportion relative to control.

### Experiment V Effects of *R. aquatilis* B301 on carbohydrate composition in phloem media after *L. procerum* growth at different time points

To provide further evidence that D-pinitol was consumed prior to D-glucose by *L. procerum* in the presence of bacteria, D-pinitol and D-glucose content in phloem media were tested at different time points. One bacterial strain, *R. aquatilis* B301, was selected. The same arena was used as in Supplementary online. Particularly, 100 μL of bacterial cultures were coated on LBA plate as treatment and LBA plate without bacteria was set as control. The phloem media were sampled at 0, 5, 10, 15, 20, 25, 30d after the fungus was inoculated, and carbohydrate composition was tested as in Supplementary online. The treatment at each point in time was replicated 6 times.

### Experiment VI Effects of D-pinitol and D-glucose on growth of *L. procerum*, RTB larvae and eight most frequently isolated bacterial strains

To compare the effects of D-glucose and D-pinitol on *L. procerum* growth, two kinds of agar media plates containing D-pinitol or D-glucose respectively were made (Supplementary online). (+)-α-pinene was also added by sticking a small glass tube full of pinene to the inside of petri dish lids. Fungi were inoculated onto the center of medium plates. Mycelial growth (linear) was measured from the point of inoculation to the leading edge of the hyphae every 2d, and along four perpendicular lines. All treatments were replicated 9 times.

To detect the effect of each single carbohydrate on weight change of RTB larvae, each pure carbohydrate (D-glucose and D-pinitol) was added back, according to their total content, into oligotrophic phloem powder in which main carbohydrates and other compositions were extracted with methanol (Supplementary online). Carbohydrate composition in oligotrophic phloem powder was tested before experiments to make sure that no D-pinitol or D-glucose was left (Supplementary, Fig. S7 online). Preliminary experiments revealed that RTB larvae can grow in both the raw phloem media and the oligotrophic phloem media (Supplementary, Fig. S8 online). RTB larvae were fed on sterile phloem media for a week, weighed (initial mass of larvae ± s.d. = 20.64 ± 3.25 mg), and then randomly assigned to each treatment which contained D-pinitol or D-glucose respectively. They were weighed again 6d later, and weight change was used to represent growth. Each treatment was replicated 56 times.

To test the effect of each single carbohydrate on growth of the most frequently isolated bacterial strains, two kinds of liquid culture media containing D-pinitol or D-glucose respectively were made (Supplementary online). 20 μL of actively growing bacterial cultures were inoculated into 4 mL of culture media. Absorbance at 600 nm was measured 12 h later and was regarded as the sole parameter to compare bacterial growth rate as the initial absorbance at 600 nm of all treatments were about 0.05. Each treatment was replicated 3 times. Bacterial strains *R. aquatilis* B301 B302 B904, *S. liquefaciens* B310 B317, *Pseudomonas* sp. 7 B321 B322 B911 were tested.

### Data analysis

Prior to statistical analysis we tested all variables for normality with the Kolmogorov-Smirnov test and homogeneity of group variances with Levene’s test. In Experiment II, RTB larva weight change was treated as dependent variable, and fungal strains or bacterial strains were treated as independent variables respectively in one-way ANOVA and followed by Tukey multiple comparisons (equal variances) or Welch’s ANOVA test followed by Dunnett’s T3 test (unequal variances). In Experiment III, fungal growth, D-glucose or D-pinitol composition was treated as dependent variable, and bacterial species as independent variables in one-way ANOVA followed by Tukey multiple comparisons. In Experiment IV, fungal growth, D-glucose or D-pinitol composition was treated as dependent variable, and different bacterial amounts were treated as independent variables in one-way ANOVA followed by Tukey multiple comparisons. In Experiment V, D-glucose and D-pinitol composition were measured at each time point for each observation (each petri dish), therefore, the observations were independent among time points. D-glucose or D-pinitol composition was treated as dependent variable, and the different points in time were treated as independent variables in one-way ANOVA and followed by Tukey multiple comparisons. For fungal growth, RTB larvae weight change, and bacterial growth in Experiment VI, data were compared by an independent-samples T test. All the data were analyzed with SPSS 18.0 (SPSS Inc., Chicago, IL, USA). Figures in this work were produced by SigmaPlot 12.5 (Systat Software Inc, San Jose, California, USA).

## Additional Information

**How to cite this article**: Zhou, F. *et al*. Altered Carbohydrates Allocation by Associated Bacteria-fungi Interactions in a Bark Beetle-microbe Symbiosis. *Sci. Rep*. **6**, 20135; doi: 10.1038/srep20135 (2016).

## Supplementary Material

Supplementary Information
