# Supplementary material for: Altered Carbohydrates Allocation by Associated Bacteria-fungi Interactions in a Bark Beetle-microbe Symbiosis
Source: Sci Rep. 2016 Feb 3;6:20135. doi: 10.1038/srep20135 (PMC4738288; doi:10.1038/srep20135)
Supplement: Supplementary Information [file srep20135-s1.pdf]

Altered Carbohydrates Allocation by Associated Bacteria-fungi Interactions in a Bark Beetle-microbe Symbiosis

Fangyuan Zhou[†, 1, 2], Qiaozhe Lou[†, 3], Bo Wang[4], Letian Xu[1, 2], Chihang Cheng[1, 2], Min Lu[1,*], Jianghua Sun[1,*]

[1]State Key Laboratory of Integrated Management of Pest Insects and Rodents, Institute of Zoology, Chinese Academy of Sciences, Beijing, 100101, China.

[2]University of Chinese Academy of Sciences, Beijing, 100049, China.

[3]Technical Center, Hebei Entry-Exit Inspection and Quarantine Bureau, Shijiazhuang, 050051, China.

[4]Key Laboratory of Tropical Forest Ecology, Xishuangbanna Tropical Botanical Garden, Chinese Academy of Sciences, Menglun, 666303, China.

[†]These authors contributed equally to the study.

* Correspondence: *E-mail:* lumin@ioz.ac.cn; sunjh@ioz.ac.cn.

Supplementary:

Figures and tables for "Altered Carbohydrates Allocation by Associated Bacteria-fungi Interactions in a Bark Beetle-microbe Symbiosis"

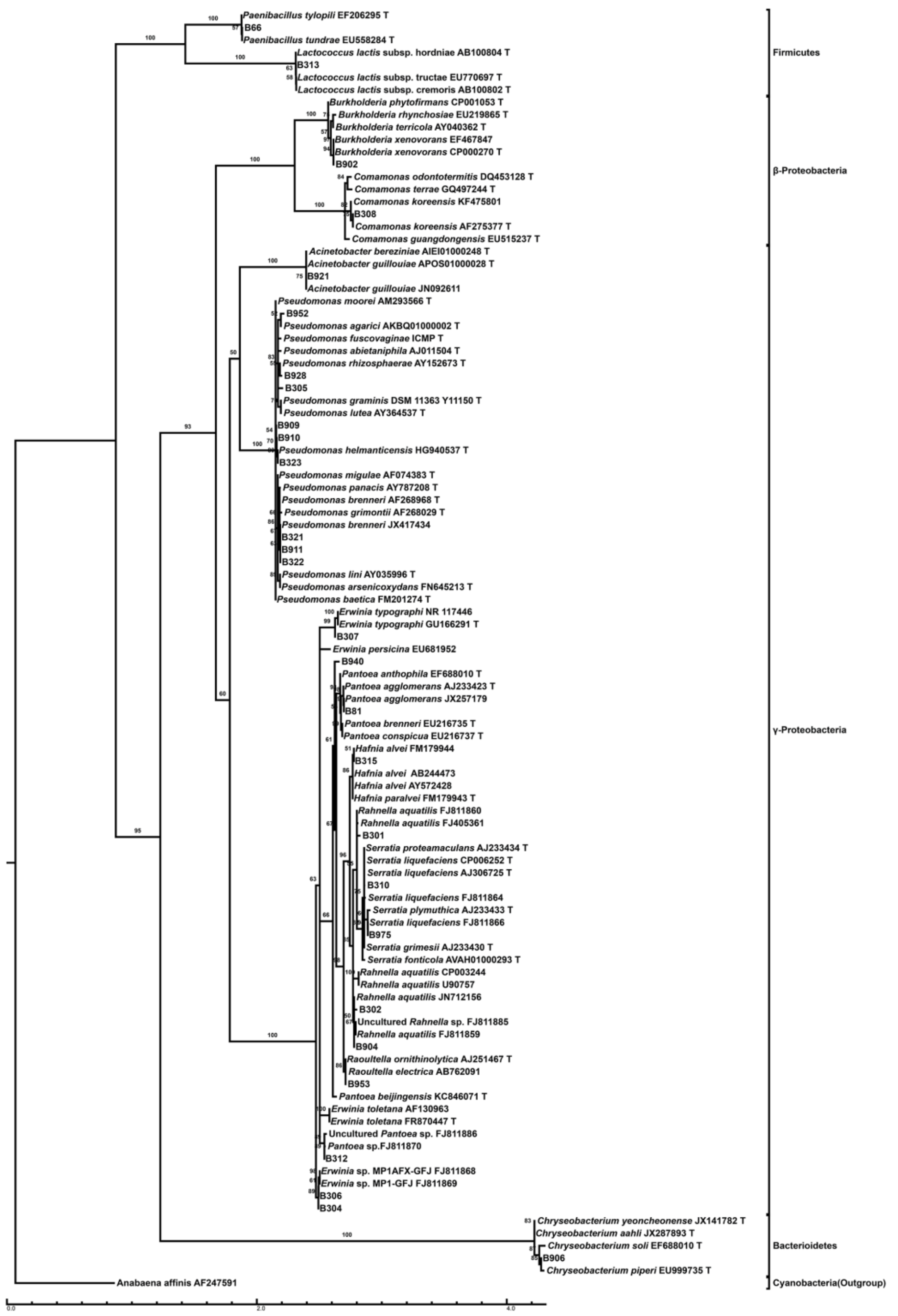

Figure S1 Maximum likelihood tree of bacterial isolates associated with Dendrotouns valens, the types (indicated with "T") and the ecologically related (indicated after the accession number with "ex." as the beginning) sequences from GenBank. The 16S rDNA sequence of *Anabaena affinis* was used as outgroup. Numbers on the nodes represent bootstrap support from 1000 replicates. Nodes with bootstrap values of 50 % or more are displayed.

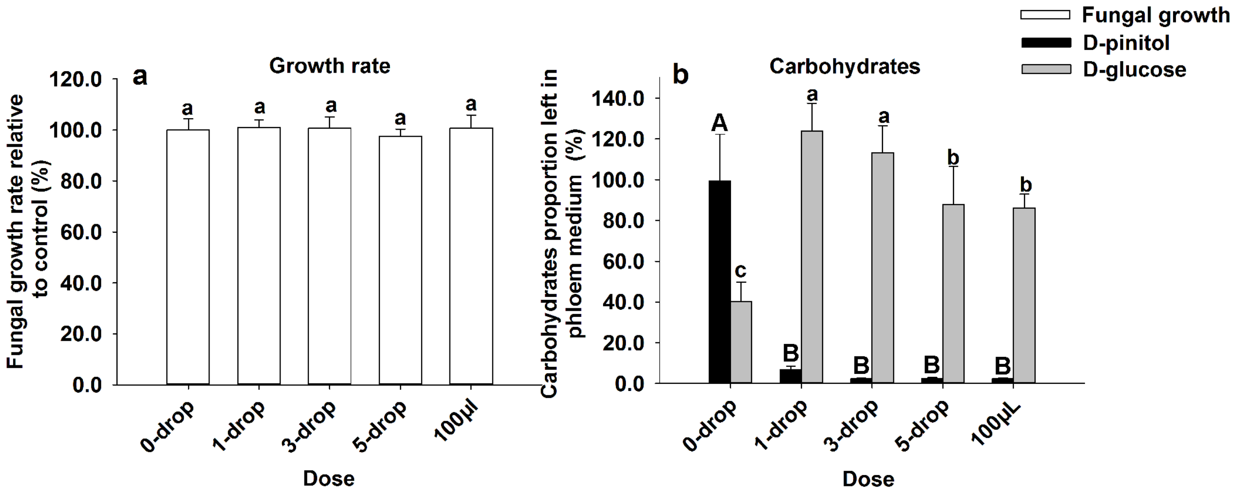

Figure S2 Effects of different amounts of *R. aquatilis* B301 culture on growth and carbohydrate consumption sequence of *L. procerum* (CMW25626) on phloem media. Fungal growth in fungus-grown phloem media was represented as change of mycelia linear growth rate relative to control (+s.e.m.). Carbohydrate consumption in fungus-grown phloem media was represented as carbohydrate content left relative to fungus-free phloem media (+s.e.m.). Different letters above each bar referred to significant difference of multiple comparisons by Tukey method within each set of bars (*capital letters* for D-pinitol in Fig. S2b, and *lowercase letters* for fungal growth in Fig. S2a and D-glucose in Fig. S2b).

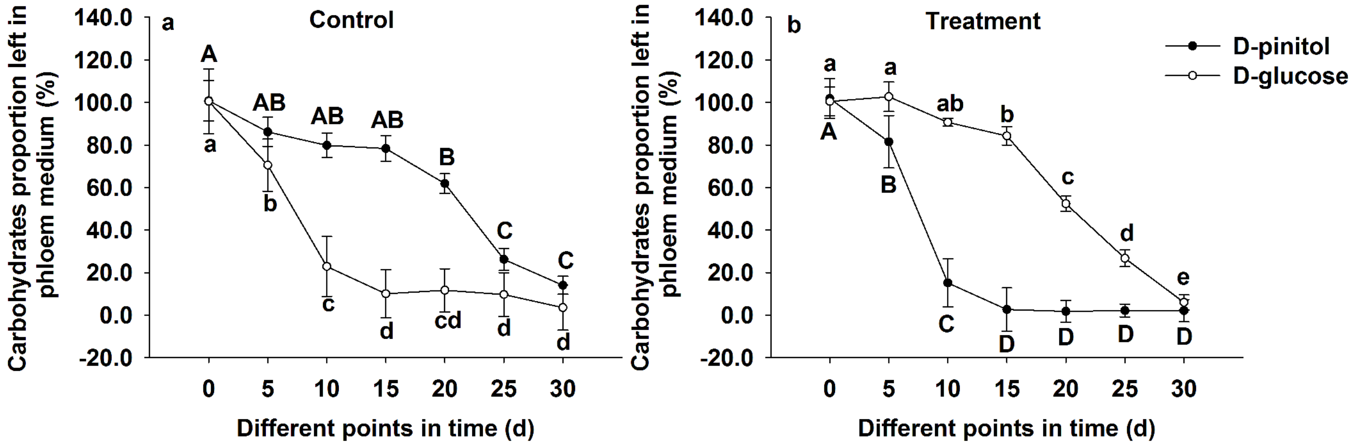

Figure S3 Carbohydrate composition in phloem media at different points in time after *L. procerum* growth with and without *R. aquatilis* B301. (a) Control group in which *R. aquatilis* B301 was not presented. (b) Treatment group in which *R. aquatilis* B301 was presented. Carbohydrate consumption in fungus-grown phloem media was represented as carbohydrate content left relative to fungus-free phloem media (+s.e.m.). Different letters above each dot referred to significant difference of multiple comparisons by Tukey method within each set of dots (*capital letters* for D-pinitol, and *lowercase letters* for D-glucose).

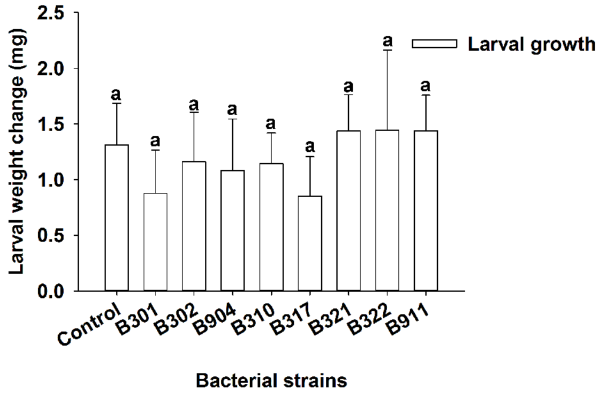

Figure S4 Effects of bacteria on growth of RTB larvae. Bacterial volatiles have no effects on RTB larvae weight change in 6d (one-way ANOVA, $F_{8, 135} = 0.298$, $p = 0.966$).

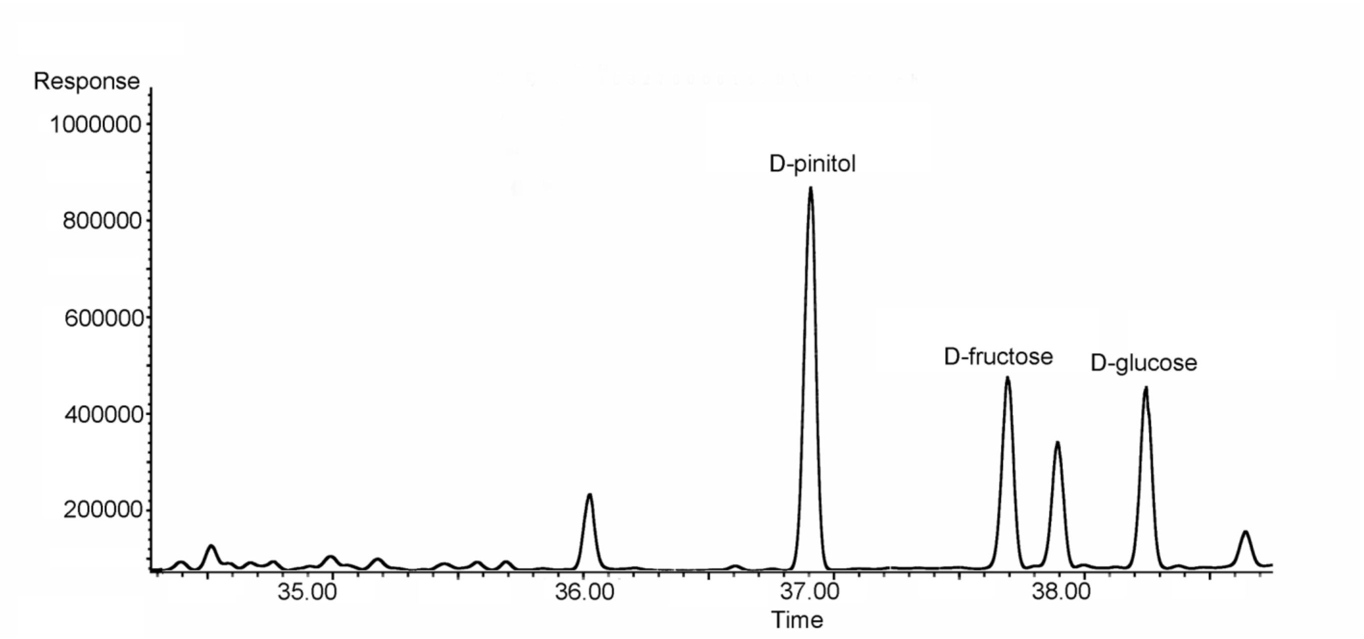

Figure S5 Gas chromatography of the carbohydrates in phloem of *Pinus tabuliformis*.

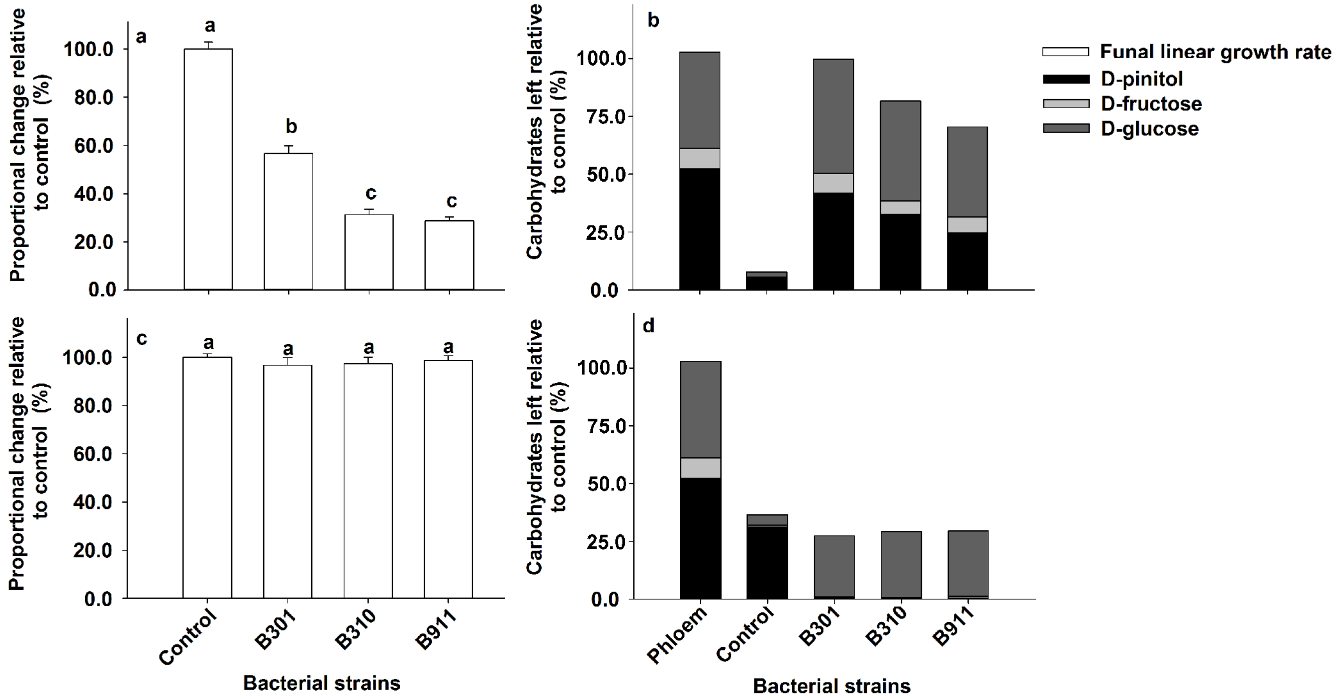

Figure S6 Effects of frequently-isolated bacteria on selected fungi growth and the carbohydrate consumption on phloem media. For fungal growth rate, different letters referred to significant difference. Growth of *O. minus* was affected by the bacterial strains significantly (Fig. S6a, one-way ANOVA, $F_{3, 16} = 839.230$, $p < 0.001$), and the consumption of D-glucose (Fig. S6b, one-way ANOVA, $F_{3, 16} = 73.362$, $p < 0.001$), D-pinitol (Fig. S6b, one-way ANOVA, $F_{3, 16} = 118.613$, $p < 0.001$) and D-fructose (Fig. S6b, one-way ANOVA, $F_{3,16} = 40.530$, $p < 0.001$) were significantly inhibited. Growth of *L. procerum* was not affected by the bacterial strains significantly (Fig. S6c, one-way ANOVA, $F_{3, 16} = 1.723$, $p = 0.203$), and significantly affected the consumption of D-glucose (Fig. S6d, one-way ANOVA, $F_{3, 16} = 6.056$, $p = 0.006$) and D-pinitol (Fig. S6d, one-way ANOVA, $F_{3, 16} = 75.629$, $p < 0.001$).

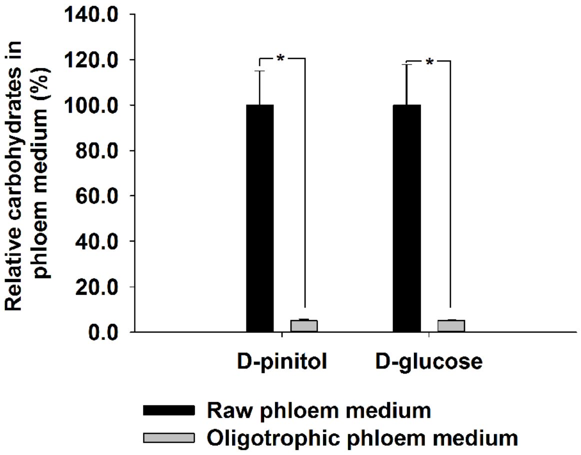

Figure S7 The carbohydrate content in oligotrophic phloem media. Content of D-glucose (Independent-sample T test, t = 5.368, df = 4.029, p <0.001) and D-pinitol (Independent-sample T test, t = 9.626, df = 4. 057, p <0.001) were significantly reduced.

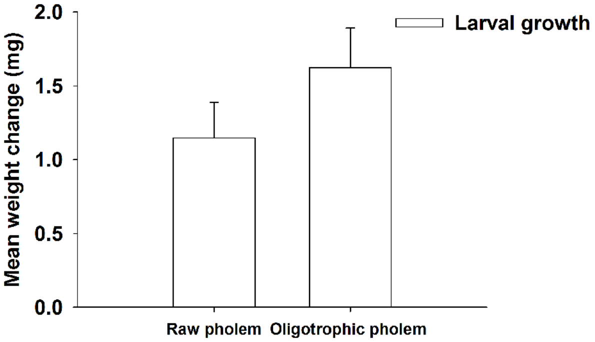

Figure S8 *Dendroctonus valens* larva weight change on raw phloem media and oligotrophic phloem media in 6d. There was no significant difference in weight change of RTB larvae in 6d on the two media (Independent-sample T test, t =-1.330, df = 110, p = 0.186).

Table S1 Frequency of isolates from different isolation sources of *Dendroctonus valens* larvae

	Bacteria	Gut						Surface						Frass						total
		1L gut	2L gut	3L gut	4L gut	5L gut	gut total	1L sur	2L sur	3L sur	4L sur	5L sur	sur total	1L fra	2L fra	3L fra	4L fra	5L fra	fra total	
**Firmicutes**	***Paenibacillus* sp.**	0	0	0	0	0	**0**	0	0	0	0	0	**0**	0	0	0	0	2	**2**	**2**
	Lactococcus lactis	0	0	0	0	2	**2**	0	0	0	0	0	**0**	0	0	0	0	0	**0**	**2**
**β-Proteobacteria**	***Burkholderia xenovorans***	0	0	0	0	0	**0**	1	0	0	0	0	**1**	0	0	0	0	0	**0**	**1**
	Comamonas koreensis	2	0	0	0	0	**2**	0	1	0	0	0	**1**	0	0	1	1	0	**2**	**5**
**γ-Proteobacteria**	***Acinetobacter guillouiae***	0	1	0	0	0	**1**	0	1	0	0	0	**1**	0	0	0	0	0	**0**	**2**
	***Pseudomonas* sp. 1**	0	0	0	1	0	**1**	1	1	0	0	0	**2**	0	0	0	0	0	**0**	**3**
	***Pseudomonas* sp. 2**	0	0	0	0	0	**0**	1	0	0	0	0	**1**	2	0	0	0	0	**2**	**3**
	***Pseudomonas* sp. 3**	1	1	0	0	2	**4**	2	1	5	1	1	**10**	0	0	4	2	0	**6**	**20**
	***Pseudomonas* sp. 4**	0	1	0	0	0	**1**	0	0	0	0	0	**0**	1	0	0	0	0	**1**	**2**
	***Pseudomonas* sp. 5**	0	0	0	1	0	**1**	0	1	1	0	0	**2**	1	1	1	1	0	**4**	**7**
	***Pseudomonas* sp. 6**	0	0	0	0	0	**0**	0	0	2	1	1	**4**	0	0	0	0	0	**0**	**4**
	***Pseudomonas* sp. 7**	0	0	2	2	10	**14**	13	7	1	3	16	**40**	0	18	20	23	19	**80**	**134**

	Hafnia alvei	0	0	0	0	0	**0**	0	4	1	0	2	**7**	0	0	0	0	0	**0**	**7**
	***Serratia* liquefaciens**	8	10	19	8	14	**59**	6	27	10	4	8	**55**	3	6	5	5	2	**21**	**135**
	Rahnella aquatilis	50	54	50	37	87	**278**	35	31	43	53	46	**208**	11	20	61	43	9	**144**	**630**
	***Raoultella* sp.**	0	0	0	1	0	**1**	1	0	0	0	0	**1**	0	0	0	0	0	**0**	**2**
	***Pantoea* sp. 1**	0	1	0	2	0	**3**	0	0	0	2	0	**2**	0	0	0	0	0	**0**	**5**
	***Pantoea* sp. 2**	0	4	0	0	0	**4**	1	1	1	6	2	**11**	0	0	0	0	0	**0**	**15**
	***Pantoea* sp. 3**	0	0	2	0	0	**2**	0	0	0	1	0	**1**	5	0	0	0	2	**7**	**10**
	***Erwinia* sp. 1**	1	1	3	0	3	**8**	1	1	1	0	0	**3**	0	0	0	0	0	**0**	**11**
	***Erwinia* sp. 2**	15	7	2	1	5	**30**	14	10	3	0	6	**33**	4	0	1	1	7	**13**	**76**
**Bacterioidetes**	***Chryseobacterium* sp.**	1	1	1	0	0	**3**	0	1	1	1	6	**9**	0	0	0	0	0	**0**	**12**
	**total**	**78**	**81**	**79**	**53**	**123**	**414**	**76**	**87**	**69**	**72**	**88**	**392**	**27**	**45**	**93**	**76**	**41**	**282**	**1088**

Table S2 GenBank accession numbers of isolates from *Dendroctonus valens* larva guts, surface and frass in China in this study and similarity scores to closest type strains and ecologically related strains in NCBI according to the 16S rDNA

Strains	GenBank accession no.	Species affiliation	Isolate source	Closest type strains	Closest type strains accession no.	Similarity (%)
**Firmicutes**						
B66	KR106592	*Paenibacillus* sp.	frass	*Paenibacillus tundrae* A10b	EU558284	99.63%(1337/1342)
B313	KR106593	*Lactococcus lactis*	gut	*Lactococcus lactis* subsp. Hordniae NCDO 2181	AB100804	99.93%(1426/1427)
**β-Proteobacteria**						
B902	KR106594	*Burkholderia xenovorans*	surface	*Burkholderia xenovorans* LB400	CP000270	99.58%(1435/1641)
B308	KR106595	*Comamonas koreensis*	gut/surface/frass	*Comamonas koreensis* KCTC 12005	AF275377	99.71%(1399/1403)
**γ-Proteobacteria**						
B921	KR106596	*Acinetobacter guillouiae*	gut/surface	*Acinetobacter guillouiae* CIP 63.46	APOS01000028	99.59%(1443/1449)
B952	KR106597	*Pseudomonas* sp. 1	gut/surface	*Pseudomonas moorei* RW10	AM293566	98.31%(1400/1424)
B928	KR106598	*Pseudomonas* sp. 2	surface/frass	*Pseudomonas rhizosphaerae* IH5	AY152673	98.34%(1420/1444)
B305	KR106599	*Pseudomonas* sp. 3	gut/surface/frass	*Pseudomonas lutea* OK2	AY364537	98.59%(1397/1417)
B909	KR106600	*Pseudomonas* sp. 4	gut/frass	*Pseudomonas baetica* a390	FM201274	99.79%(1432/1435)
B910 B958 B960 B971 B976 [a]	KR106601-KR106605	*Pseudomonas* sp. 5	gut/surface/frass	*Pseudomonas baetica* a390	FM201274	99.58%(1628/1634)
B323	KR106606	*Pseudomonas* sp. 6	surface	*Pseudomonas helmanticensis* OHA11	HG940537	99.86%(1415/1417)
B321	KR106607	*Pseudomonas* sp. 7	gut/surface/frass	*Pseudomonas brenneri* CFML 97-391	AF268968	99.58%(1410/1416)
B911 B908	KR106608-KR106609	*Pseudomonas* sp. 7	gut/surface/frass	*Pseudomonas brenneri* CFML 97-391	AF268968	99.44%(1408/1416)
B322	KR106610	*Pseudomonas* sp. 7	gut/surface/frass	*Pseudomonas brenneri* CFML 97-391	AF268968	99.65%(1411/1416)
B315	KR106611	*Hafnia paralvei*	surface	*Hafnia paralvei* ATCC 29927	FM179943	99.72%(1414/1418)
B310	KR106612	*Serratia liquefaciens*	gut/surface/frass	*Serratia liquefaciens* ATCC 27592	CP006252	99.71%(1417/1421)

B975 B916 B917 B961 B962 B963 B965 B314	KR106613-KR106620	*Serratia liquefaciens*	gut/surface/frass	*Serratia proteamaculans* DSM 4543	AJ233434	99.37%(1415/1424)
B301 B915 B918 B919 B927 B930 B933 B934 B938 B943 B945 B946 B956 B964 B966 B972	KR106621-KR106636	*Rahnella aquatilis*	gut/surface/frass	*Serratia grimesii* DSM 30063	AJ233430	98.31%(1399/1423)
B904 B924 B926 B935 B939	KR106637-KR106641	*Rahnella aquatilis*	gut/surface/frass	*Serratia plymuthica* DSM 4540	AJ233433	98.45%(1398/1420)
B302 B901 B903 B905 B913 B937 B941 B942 B947 B951 B955	KR106642-KR106652	*Rahnella aquatilis*	gut/surface/frass	*Serratia plymuthica* DSM 4540	AJ233433	98.03%(1392/1420)
B953	KR106653	*Raoultella* sp.	gut/surface	*Raoultella ornithinolytica* JCM 6096	AJ251467	99.19%(1436/1448)
B81 B309	KR106654-KR106655	*Pantoea* sp. 1	gut/surface	*Pantoea agglomerans* DSM 3493	AJ233423	99.79%(1420/1423)
B940 B973	KR106656-KR106657	*Pantoea* sp. 2	gut/surface	*Pantoea wallisii* LMG 26277	JF295057	97.61%(1309/1341)
B312	KR106658	*Pantoea* sp. 3	gut/surface/frass	*Erwinia toletana* CECT 5263	FR870447	97.11%(1379/1420)
B307 B914 B944 B977	KR106659-KR106662	*Erwinia* sp. 1	gut/surface	*Erwinia typographi* DSM 22678	GU166291	99.13%(1374/1386)

B306 B923 B950 B974	KR106663-KR106666	*Erwinia* sp. 2	gut/surface/frass	*Erwinia psidii* LMG 7034	Z96085	98.63%(1373/1418)
B304	KR106667	*Erwinia* sp. 2	gut/surface/frass	*Erwinia psidii* LMG 7034	Z96085	98.63%(1373/1418)
**Bacterioidetes**						
B906	KR106668	*Chryseobacterium* sp.	gut/surface	*Chryseobacterium piperi* CTM	EU999735	98.37%(1387/1410)

“[a]” indicates that all these strains share identical 16S rDNA sequence

Table S3 Dominant carbohydrates (D-glucose, D-pinitol and D-fructose) content in phloem of healthy *Pinus tabuliformis* Carrière

	Carbohydrate（mg/g, DW）
D-pinitol	21.96±4.10
D-fructose	14.82±3.68
D-glucose	18.12±6.65
Total	54.89±13.29

Detailed materials and methods for "Altered Carbohydrates Allocation by Associated Bacteria-fungi Interactions in a Bark Beetle-microbe Symbiosis"

Organisms, media and chemicals

Leptographium procerum (CMW25626) and *Ophiostoma minus* (CMW26254) used in this paper were from stock collections of the Forestry and Agricultural Biotechnology Institute, University of Pretoria preserved by Lu Min. RTB larvae for bioassays were obtained through the method previously described[1]. Specifically, adults of *Dendroctonus valens* collected from Tunlanchuan Forestry Farm (N 37º48′, E 111º44′; average elevation 1400 m, Shanxi, China) were introduced into newly cut bolts of *Pinus tabuliformis* Carrière in pairs, and offspring RTB larvae were collected 35 days later.

Phloem samples were collected from trunks (0.5-1 m above the ground) of healthy *P. tabuliformis* Carrière in Tunlanchuan Forestry Farm, and these trees' DBHs (diameter at breast height) were 33 to 43 cm. The phloem samples were put into bags and placed on ice, then they were transferred to the lab and weighed before vacuum freeze-dried at -20 ℃ for 48h. Dried samples were weighed again to calculate water content, and then they were grounded to phloem powder with a pulp refiner.

Phloem media (phloem powder 20 g, agar 10 g, water 300 mL) were made by the method previously described[1]. LB Broth with agar was purchased from Sigma/Aldrich (Shanghai, China). Yeast extract and select agar were purchased from BD (Becton, Dickinson and Company).

Nystatin (CAS: 1400-61-9) was purchased from Amresco, and cycloheximide (CAS:

66-81-9) was purchased from Solarbio. (+)-α-pinene (CAS: 7785-70-8), 2,2,4-Trimethylpentane (CAS: 540-84-1), chloroform (CAS: 865-49-6), pyridine (CAS: 110-86-1), D-pinitol (CAS: 10284-63-6), D-glucose (CAS: 50-99-7), D-fructose (CAS: 57-48-7), and ribitol (CAS: 488-81-3) were purchased from J&K Scientific Ltd. MSTFA (N-Methyl-N-(trimethylsilyl)trifluoroacetamide, CAS: 24589-78-4) and methoxyamine hydrochloride (CAS: 593-56-6) were purchased from Sigma/Aldrich (Shanghai, China).

Bacteria isolation and identification

Sample collection: RTB larvae and frass were collected from Beishe Mountain and Laoyagou Mountain (about 13 km far from each other) in Tunlanchuan Forestry Farm (N 37º48′ E 111º44′, average elevation 1400 m, Shanxi, China). About 15 attacked pine stumps (at least 200 m far from each other) were randomly selected for RTB larvae and frass sampling. In total, we sampled 132 RTB larvae and 26 frass samples. All the samples were stored in ice boxes and taken back to laboratory. Larvae were grouped by their developmental stages roughly according to their head capsule size[2].

Bacteria isolation through culture-dependent method: Bacteria were isolated through a culture-dependent method described previous[3]. To isolate the bacteria from larval body surface and frass, larva and frass samples were immersed in 200 μL of 10% PBS respectively, sonicated for 1 min and vortexed for 10 s, then 100 μL of the suspension (diluted from 10^{-2} to 10^{-6}) were plated on LBA containing nystatin (40 mg/L) and cycloheximide (0.5 mg/L). To isolate bacteria from guts, individual guts of 3rd to 5th instar RTB larvae were obtained by dissection of

surface-sterilized larvae, and guts of 1st and 2nd instar larvae were replaced by surface-sterilized larva bodies as these larvae were too small for dissection. Gut samples were crushed and plated as described above. After the plates were incubated at 28 °C for 12-48 h, colonies from each plate were counted, and selected to streak on LBA plates for purification. Pure cultures were morphologically categorized and counted according to their size, color, thickness, transparency, and texture. Bacterial strains isolated from at least two different samples in each group were selected for 16S rDNA sequencing.

Bacterial DNA extraction, PCR and sequence for 16S rDNA: Bacterial DNA was extracted using a blood cell DNA extraction kit (Saibaisheng, China) after 1 h of digestion at 30 ℃ by 4 mg/mL lysozyme (Tiangen, China) in 500 µL 50 mM EDTA buffer (pH 8.0). 16S rDNA genes were amplified with primers as follows[4]:

1492 R (5’-GGCTCGAGCGGCCGCCCGGGTTACCTTGTTACGACTT-3’).

8F (5’-GCGGATCCGCGGCCGCTGCAGAGTTTGATCCTGGCTCAG-3’)

The reaction mixture contained 1.2 µL of dNTPs (10 mM/each), 5 µL of 10 × PCR buffer (with $MgCl_2$), 2 µL of each primer (10 µM/each), 0.8 µL LA Taq polymerase (5 U/µL) (TaKaRa, China), and 10-100 ng of DNA. Then the mixture was adjusted to 50 µL with sterilized deionized water. The reaction conditions were 94 ℃ for 5 min, 35 cycles of 30 s at 94 ℃, 30 s at 51 ℃, and 1 min 30 s at 72 ℃ with a final extension at 72 ℃ for 10 min. Expected PCR products (1500 bp) were sequenced in two directions on an ABI 3730XL DNA Analyzer (Applied Biosystems, USA) with the same primers. Consensus sequences were manually assembled and

edited according to chromatograms in MEGA5[5].

Phylogenetic analysis of selected 16S rDNA sequences: All 16S rDNA sequences were aligned online with EzBioCloud database (http: //www. eztaxon-e.ezcloud.net/)[6] and BLAST search (http://blast.ncbi.nlm.nih.gov/Blast.cgi). Sequences in this study were uploaded in the GenBank database (supplementary, Table S2). Besides, the 16S rDNA sequences of the selected isolates were phylogenetically analyzed. Besides, sequences of the closest type strains and ecologically related strains downloaded from the databases mentioned above were added and aligned using Clustal X[7], followed by manual refinement in BIOEDIT[8]. *Anabaena affinis* (AF247591) was used as outgroup. We used the software jModeltest 2.1[9] to select Nucleotide substitution model based on Akaike Information Criterion[10]. RAXML (version 7.4.2) was used to reconstruct the phylogenetic relationship using maximum likelihood method[11], and confidence at each node was assessed by 1,000 bootstrap replicates[12]. The phylogeny tree was visualized and edited in TreeGraph 2[13].

GC-MS and GC-FID analyses for dominant carbohydrates (D-glucose, D-pinitol and D-fructose) in phloem of healthy *P. tabuliformis* Carrière and in phloem media

Eleven phloem powder samples, each from one single tree, and phloem media were subjected to GC-FID analyses to test carbohydrate composition by the method described by Lisec with little modification[14]. 200 mg samples and 5 mL 100% methanol were put into a 10 mL centrifuge tube and shaken for 20 min at 70 ℃ in a thermomixer at 950 r.p.m. After that the samples were centrifuged for 10 min at 11000 g, and 350 μL supernatant with 80 μL 0.2 mg/mL

ribitol as an internal quantitative standard were transferred into 350 μL 100% methanol. With 300 μL chloroform and 600 μL dH_2O added, the samples were vortexed for 30s and centrifuged at 2200 g for 20 min, and 150 μL of supernatant were transferred to a new 1.5 mL centrifuge tube. After the extracts were dried in a vacuum container, 40 μL of methoxyamination reagent were added into the samples and the mixtures were shaken at 37 ℃ for 3 h. Then 70 μL of MSTFA reagent were added and shaken at 37 ℃ for 1.5 h. At last, 300 μL dH2O and 500 μL isooctane were added into the samples and vortexed for 30 s. The supernatants were filtered with sodium sulphate anhydrous into a GC-MS glass vial and analyzed by GC-MS for compound identification and GC-FID for quantification.

Identification analysis was carried on GC (Aglient 6890N) and MSD (Aglient 5973). Quantification analysis was carried on GC (Aglient 7890A) and FID (flame ionization detector). Standard carbohydrates (D-glucose, D-pinitol and D-fructose) were also tested by GC-FID to check the retention time. Mass conditions were as follows: injection volume 1 μL without split, helium as carrier gas at 1.0 mL/min constant flow mode, injector temperature 230 ℃, HP-5 silica capillary column (60 m×0.25 mm×0.25 μm). Oven temperature program was isothermal for 5 min at 70℃, followed by a 5 ℃ per min ramp to 310 ℃， and holding at this temperature for 12 min. The relative percentages of the individual components were calculated based on GC peak area (FID response) without using correction factors.

Preliminary experiment: Effects of frequently-isolated bacteria on selected fungi growth and carbohydrate consumption on phloem media

To test whether the associated bacteria could inhibit fungal growth and result in reduction of carbohydrate consumption, a preliminary experiment was conducted with only three bacterial strains (*Rahnella aquatilis* B301, *Serratia liquefaciens* B310, and *Pseudomonas* sp7 B321) and the two selected fungi (*L. procerum* and *O. minus*). The experiment was conducted by the method described by Adam[15]. (+)-α-pinene was added into the arena to simulate host tree compounds. Briefly, LB agar was poured into one side of a 90 mm petri dish which was separated into two parts. About 1 cm wide LB agar approximately was removed along the division of each dish to make space for a glass tube containing autoclaved cotton soaked by 200 μL of 99.0% (+)-α-pinene. 100 μL of actively growing bacterial cultures in LB was inoculated onto LBA and spread with a glass spreading rod. Phloem media were poured into the opposite side of the petri dish for fungus growth. One plug of agar (3 mm diameter) from the leading edge of fungi culture growing on MEA was inoculated onto the phloem medium plate. The plug was placed about 1 cm from the division of the dish, and about 1 cm away from the exterior edge of the dish. Fungal linear growth along the plastic division was measured from the point of inoculation to the leading edge of the hyphae every 2d, and LBA plates without bacteria were regarded as control, and all treatments were replicated 5 times. Fungal linear growth rate from each bacterial treatment and control were compared with ANOVA by Tukey in SPSS 18.0 (SPSS Inc., Chicago, IL, USA).

Carbohydrate composition left in the phloem media with fungal isolate was detected by the method of Lisec[14] with modification. For each petri dish in this experiment, phloem media were

collected to quantify left carbohydrate composition after the fungi grew to cover 100% of the medium (The fungus grew to the edge of the dish at about 10d, and the phloem media were usually collected at 15d to make sure that the hyphae covered 100% of the medium surface). Carbohydrate composition left in the phloem medium from each bacterial treatment was compared with one-way ANOVA by Tukey in SPSS 18.0 (SPSS Inc., Chicago, IL, USA).

Culture media for testing the effects of D-pinitol and D-glucose on growth of L. procerum, RTB larvae and frequently-isolated bacteria

For *L. procerum*, we chose D-pinitol or D-glucose as single carbon source by the method previously described[16]. The basal medium contained select agar 20 g/L, KH_2PO_4 1 g/L, $MgSO_4 \cdot 7H_2O$ 0.2 g/L, $FeCl_3 \cdot 6H_2O$ 0.02g/L, and yeast extract 1 g/L. Carbohydrate solutions sterilized by filtration (0.22 μm) were added into the basal medium to make the final concentration of carbohydrate 0.4%.

For RTB larvae, oligotrophic phloem powder was made by extracting all carbohydrates and other compositions with methanol. Specifically, 50g of phloem powder was put into 200 mL of methanol and shaken at 70 ℃ for 30 min. Then it was filtered by 5 layers of gauze before cooling. The phloem powder was treated three times as described above and dried before further experiments. Two kinds of media were prepared by the method previously described[1] with modification and as follows: (A) D-glucose: 6.07% oligotrophic phloem powder, 3.03% agar and 0.3% D-glucose; (B) D-pinitol: 6.07% oligotrophic phloem powder, 3.03% agar and 0.3% D-pinitol. All those two mediums were autoclaved (121℃, 15min, 0.105 Mpa) and poured about

4 mL into 30 mm petri dishes for further experiments.

For frequently-isolated bacteria, we chose M9 as the basal medium and one kind of carbohydrate was added as the sole carbon source in liquid culture media. Specifically, carbohydrates dissolved in dH_2O were sterilized by filtration (0.22 μm). M9, 1 M $MgSO_4$, and 1 M $CaCl_2$ were autoclaved separately (121℃, 15min, 0.105 Mpa). An aliquot of the carbohydrate solution were transferred into the M9 to make the final concentration of carbohydrates 1%. $MgSO_4$ and $CaCl_2$ solution were added to a final concentration of 2 mM and 0.1 mM respectively after cooling. (+)-α-pinene was solved in DMSO and added into the above culture media to a final concentration of 0.5 mg/mL.

Non-contact effects of associated bacteria on growth of RTB larvae

The same arena as described above was used to test whether associated bacteria could influence the weight change of RTB larvae in non-contact ways during the 6d periods. However, the difference was that one RTB larva was put into the phloem media instead of fungi. RTB larvae were fed on sterile phloem medium for a week, weighed (initial mass of larvae ± s.d. = 19.33 ± 4.15 mg), and then randomly assigned to each treatment. Bacterial strains *R. aquatilis* B301 B302 B904, *S. liquefaciens* B310 B317, and *Pseudomonas* sp7 B312 B322 B911 were selected, and LBA plate without bacteria was used as control. Each treatment was replicated 16 times. RTB larvae weight change during the 6 days of each bacterial treatment was compared with control by an independent-samples T test with SPSS 18.0 (SPSS Inc., Chicago, IL, USA).

Effects of fungus-colonized phloem media in the presence or in the absent of bacteria

during fungal growth on RTB larval growth

To test whether the bacterial strains could alleviate or compromise the antagonistic effects from fungi *O. minus* and *L. procerum* on RTB larval growth, we first investigated the effects of fungus-colonized phloem media on RTB larval growth according to the method described previously [1]. Particularly, the two fungal strains were inoculated on phloem media and incubated at 25 ℃, RH 70 percent in darkness until the mycelia covered all the media (about 10d). (+)-α-pinene was also added by sticking a small glass tube full of pinene to the inside of petri dish lids. Subsequently, 3 cm diameter media discs were made and transferred into 30 mm petri dishes. RTB larvae (initial mass of larvae ± s.d. = 20.72 ± 6.01 mg) were fed on medium with specific fungal strain and without fungal strain (control), and they were weighed after 6 days. The weight change was used to represent growth. Each treatment had 36 replicates. Secondly, effects of fungus-colonized phloem media in the presence or in the absent of bacteria on RTB larval growth were detected. In particular, fungal strains of *O. minus* and *L. procerum* were inoculated on phloem media in the presence or absence of bacterial strains with (+)-α-pinene also added. For each fungal strain, the fungus-colonized media was regarded as control media, while the phloem media which were colonized by fungus in the presence of all eight bacterial strains (*R. aquatilis* B301 B302 B904, *S. liquefaciens* B310 B317, *Pseudomonas* sp. 7 B321 B322 B911) were regarded as different treatments. After the mycelia covered all the media, these fungus-colonized phloem media were used to feed RTB larvae (initial mass of larvae ± s.d. = 21.36 ± 6.00 mg for *O. minus*, 21.38 ± 6.02 mg for *L. procerum*) to test their effects on larval

growth as described above. Each treatment had 40 replicates.

References

1 Wang, B., Lu, M., Cheng, C., Salcedo, C. & Sun, J. Saccharide-mediated antagonistic effects of bark beetle fungal associates on larvae. *Biol. Lett.* **9,** 20120787 (2013).

2 Liu, Z., Xu, B. & Sun, J. Instar numbers, development, flight period, and fecundity of *Dendroctonus valens* (Coleoptera: Curculionidae: Scolytinae) in China. *Ann. Entomol. Soc. Am.* **107,** 152-157 (2014).

3 Lou, Q. Z., Lu, M. & Sun, J. H. Yeast diversity associated with invasive *Dendroctonus valens* killing *Pinus tabuliformis* in China using culturing and molecular methods. *Microb. Ecol.* **68,** 397-415 (2014).

4 Weisburg, W. G., Barns, S. M., Pelletier, D. A. & Lane, D. J. 16S ribosomal DNA amplification for phylogenetic study. *J. Bacteriol.* **173,** 697-703 (1991).

5 Tamura, K. *et al.* MEGA5: molecular evolutionary genetics analysis using maximum likelihood, evolutionary distance, and maximum parsimony methods. *Mol. Biol. Evol.* **28,** 2731-2739 (2011).

6 Kim, O. S. *et al.* Introducing EzTaxon: a prokaryotic 16S rRNA Gene sequence database with phylotypes that represent uncultured species. *Int. J. Syst. Evol. Micr* **62,** 716-721 (2012).

7 Thompson, J. D., Gibson, T. J., Plewniak, F., Jeanmougin, F. & Higgins, D. G. The

CLUSTAL_X windows interface: flexible strategies for multiple sequence alignment aided by quality analysis tools. *Nucleic. Acids. Res.* **25,** 4876-4882 (1997).

8 Hall, T. A. BioEdit: a user-friendly biological sequence alignment editor and analysis program for Windows 95/98/NT. *Nucleic acids symposium series* **41,** 95-98 (1999).

9 Darriba, D., Taboada, G. L., Doallo, R. & Posada, D. jModelTest 2: more models, new heuristics and parallel computing. *Nat. Methods* **9,** 772-772 (2012).

10 Posada, D. & Buckley, T. R. Model selection and model averaging in phylogenetics: Advantages of akaike information criterion and bayesian approaches over likelihood ratio tests. *Syst. Biol.* **53,** 793-808 (2004).

11 Stamatakis, A., Ludwig, T. & Meier, H. RAxML-III: a fast program for maximum likelihood-based inference of large phylogenetic trees. *Bioinformatics* **21,** 456-463 (2005).

12 Hillis, D. M. & Bull, J. J. An empirical test of bootstrapping as a method for assessing confidence in phylogenetic analysis. *Syst. Biol.* **42,** 182-192 (1993).

13 Stöver, B. C. & Müller, K. F. TreeGraph 2: combining and visualizing evidence from different phylogenetic analyses. *BMC bioinformatics* **11,** 7 (2010).

14 Lisec, J., Schauer, N., Kopka, J., Willmitzer, L. & Fernie, A. R. Gas chromatography mass spectrometry-based metabolite profiling in plants. *Nat. Protoc.* **1,** 387-396 (2006).

15 Adams, A. S., Currie, C. R., Cardoza, Y., Klepzig, K. D. & Raffa, K. F. Effects of symbiotic bacteria and tree chemistry on the growth and reproduction of bark beetle

fungal symbionts. *Can. J. Forest. Res.* **39,** 1133-1147 (2009).

16 Sati, S. C. & Bisht, S. Utilization of various carbon sources for the growth of waterborne conidial fungi. *Mycologia* **98,** 678-681 (2006).
